# The influence of implant position and of prosthetic characteristics on the occurrence of peri-implantitis: a retrospective study on periapical radiographs

**DOI:** 10.1007/s00784-023-05303-9

**Published:** 2023-11-01

**Authors:** Stefano Corbella, Benedetta Morandi, Elena Calciolari, Alice Alberti, Luca Francetti, Nikolaos Donos

**Affiliations:** 1 Department of Biomedical, Surgical and Dental Sciences, Università degli Studi di Milano, Milan, Italy; 2 IRCCS Ospedale Galeazzi Sant’Ambrogio, Milan, Italy; 3 Centre for Oral Clinical Research, Institute of Dentistry, Faculty of Medicine and Dentistry, Queen Mary University of London, London, UK; 4 Centro di Odontoiatria, Dipartimento di Medicina e Chirurgia, Università di Parma, Parma, Italy

**Keywords:** Dental implants, Peri-implantitis, Risk factors, Dental radiography, Dental prosthesis design

## Abstract

**Objective:**

The present retrospective study aimed to investigate the influence of malposition on the occurrence of peri-implantitis.

**Materials and methods:**

The study included clinical records of systemically healthy patients with single and partial implant-supported rehabilitations and at least 1-year post-loading follow-up. The parameters collected included implant-related factors, patient-related factors, site-related factors, and prosthesis-related factors. The radiographic measurements were taken by using a dedicated software and the diagnosis of peri-implantitis was made based on all the available clinical and radiographic data. Descriptive statistics were provided for all variables. Following an exploratory approach, an implant-level analysis of factors influencing the occurrence of peri-implantitis was done through a multilevel multivariate logistic regression (mixed).

**Results:**

A total of 180 implants belonging to 90 subjects were randomly selected. Malposition showed no statistically significant association with the occurrence of peri-implantitis. According to the multi-level analysis, the parameters that were significantly associated with peri-implantitis included presence / history of periodontitis (OR = 5.945, 95% CI: 1.093 – 32.334, *P* = 0.039) and presence of an emergence profile angle ≥ 45° (OR = 9.094, 95% CI: 2.017 – 40.995, *P* = 0.005).

**Conclusions:**

Implant malposition, as defined following Buser’s criteria (2004), did not influence the occurrence of peri-implantitis in the selected cohort. Conversely, history of periodontitis and presence of a prosthetic emergence profile with an angle ≥ 45° were correlated to an increased risk of peri-implantitis.

## Introduction

Dental implants are widely adopted for the rehabilitation of partial and full edentulism, being supported by solid scientific evidence that demonstrated their stability over time and high survival rates [1–3]. Nevertheless, implant loss could occur at different timepoints, distinguishing between early implant loss, that happens as a failure in the osseointegration process, and late implant loss, which is correlated with the occurrence of a late — biological or technical — complication [4]. Peri-implantitis is the most common biological complication that may cause implant failure over time [4], and it is characterized by signs of peri-implant tissue inflammation, radiographic evidence of bone loss, and presence of peri-implant probing that has increased over time [5].

Peri-implantitis is a highly prevalent disease, as suggested by several epidemiological studies. One recent article on electronic records in the United States (2127 patients / 6129 implants) found that, over an average follow-up period of 2 years, 34% of patients and 21% of implants presented with peri-implantitis [6]. Another study on a European population consisting of 596 implants in 62 patients (Swedish) found that 45% of all patients showed peri-implantitis 9 years after implant treatment [7]. Two studies of our research group explored the prevalence of peri-implantitis through the analysis of single, partial, and full-arch restorations revealing that, after 5 years, the 4.6% of implants and 12.7% of patients with full-arch restorations showed peri-implantitis [8, 9]. However, as reported in a systematic review of the literature on 15 studies, the prevalence of peri-implantitis is very heterogeneous and could range between 1.1 and 85% at implant level and 26% (median) at patient level, with implants having more than 5 years follow-up [10]. One recent study notably observed that the new diagnostic criteria based on the recent classification reduced the measure of the prevalence of peri-implantitis (both implant- and patient-level), as compared to prior analyses, bringing new risk factors into focus [11].

The prevention and management of peri-implantitis should be based on a deep understanding of which are the most important risk factors for the disease [12]. History of periodontitis and inadequate level of oral hygiene are well-known risk factors for peri-implantitis [13, 14].

In recent years, some studies have focused on assessing if different implant- and prosthesis-related factors could increase the risk of developing peri-implantitis [15, 16]. Despite pre-clinical studies seem to suggest that implant surface characteristics may have a relevant role on peri-implantitis progression, a recent consensus concluded that there is no clear scientific evidence that such characteristics could have a significant impact [17]. The same consensus also highlighted the outcomes of two studies indicating that an emergence angle of the prosthesis of more than 30° with a convex profile is associated to an increased risk of peri-implantitis, mainly due to the difficulties in maintaining oral hygiene [18, 19]. The same conclusions were confirmed by one recently published systematic review of the literature [16]. On the contrary, another recent review that included four studies did not confirm that emergence angle (higher or lower than 30°) may have an influence on peri-implant bone resorption rate, however affirming that a convex profile can be associated to peri-implantitis [15].

Despite it is biologically plausible to think that implant malposition can significantly influence the development of peri-implantitis, its effect has been scarcely studied in the literature, also because of the difficulties in defining “malposition” itself. One study, not designed to answer this specific question, suggested a significant effect also of implant position (or better malposition) on the outcomes of implant therapy [20]. On the other end, another systematic review of the literature affirmed that surgical experience does not influence the outcome of implant treatment [21]. Interestingly, one recent paper highlighted that peri-implantitis may result also because of errors made by the clinician in implant therapy, including errors in patients’ selection or related to wrong implant placement [22]. The scarcity of data about how implant position and characteristics of the prosthesis may influence the occurrence of peri-implantitis represents the rationale of the present research.

Therefore, the aim of the study was to investigate whether implant malposition may influence the occurrence of peri-implantitis. The secondary aim is to investigate the influence of the collected implant-related, patient-related, and prothesis-related characteristics on the same outcome, by analyzing the factors influencing them. The null hypothesis is that implant malposition does not modify the occurrence of peri-implantitis.

## Materials and methods

### Study design

This is a retrospective study, whose protocol obtained the approval of the Ethical Committee of the IRCCS Ospedale San Raffaele in Milan, Italy (37/INT/2022). All the phases of the study were carried out following the principles of the Helsinki Declaration for Research on Human Subjects [23]. The study was reported following the indications included in the “Strengthening the Reporting of Observational studies in Epidemiology (STROBE)” guidelines [24]. The data retrieved were all anonymized.

### Settings and participants

The clinical and radiographic records of all subjects treated with implant-supported rehabilitations in the Dental Clinic of the IRCCS Istituto Ortopedico Galeazzi in Milan, Italy, in the period that ranged between January 1 2005, and June 1 2021 were screened for inclusion by applying the following criteria: (i) the radiographs and clinical records must belong to ≥ 18-year-old subjects at the time of implant placement; (ii) records of subjects who gave written informed consent for using radiographs and data for research purposes (in anonymized form); (iii) being referred to subjects treated with implants with a moderately rough surface, with single and partial rehabilitations not immediately-loaded and with at least 1-year post-loading of follow-up (without cantilever extension), included in a maintenance program with yearly recalls; (iv) single-tooth restorations (single implants with at least one adjacent tooth) and multiple tooth restorations (one tooth per implant or bridges, splinted or not); (iv) being of subjects without any systemic disease that could have an impact on bone metabolism (e.g., diabetes mellitus, osteoporosis, neoplasms).

We excluded records with (i) incomplete information about patient status (systemic diseases, smoking status, medications, age, gender) and incomplete description of the surgical and prosthetic protocol that was adopted; (ii) incomplete information to assess periodontal status at the time of intervention; (iii) without at least one periapical radiograph of good quality taken at the time of prosthetic loading and an insufficient number of follow-up visits (at least one per year); (iv) implants belonging to full-arch restorations.

The quality of the images were assessed by the Guidance Notes for Dental Practitioners on the Safe Use of X-Ray Equipment, accepting Grade 1 and Grade 2 images [25].

### Outcome variables and data collection

The primary outcome of the study was the occurrence of peri-implantitis, which was defined following the criteria by Berglundh et al. [5] and which required the presence of signs of inflammation (bleeding and / or suppuration after probing) and radiographic bone loss beyond crestal bone resorption due to initial remodeling. Whenever the 1-year radiograph was missing, peri-implantitis was defined based on the presence of bone level ≥ 3 mm apically to the most coronal portion of the intraosseous portion of the implant body, and on an increasing probing depth as compared to previous measurements (if available). In case of multiple implants with peri-implantitis, we considered the implant with the shortest follow-up as the first occurrence of the disease.

For the purposes of this study, the diagnosis of peri-implantitis was made based on all the available clinical and radiographic data, and the time of the diagnosis was considered as censoring time and maximum follow-up for such implant. For healthy implants the last follow-up time was recorded as the time of the last radiographic and clinical control visit.

Implant malposition was defined when the implant under analysis did not follow even one of these criteria proposed by Buser and coworkers in 2004 [26]:

(i) at least 1.5 – 2 mm between implant neck and adjacent tooth (mesio-distal); ii) at least 1 mm of apico-coronal distance between implant neck and the cemento-enamel junction (CEJ) of adjacent teeth (no more than 2.5 mm); (iii) at least 3 mm between the necks of two adjacent implants.

**Fig. 1:**
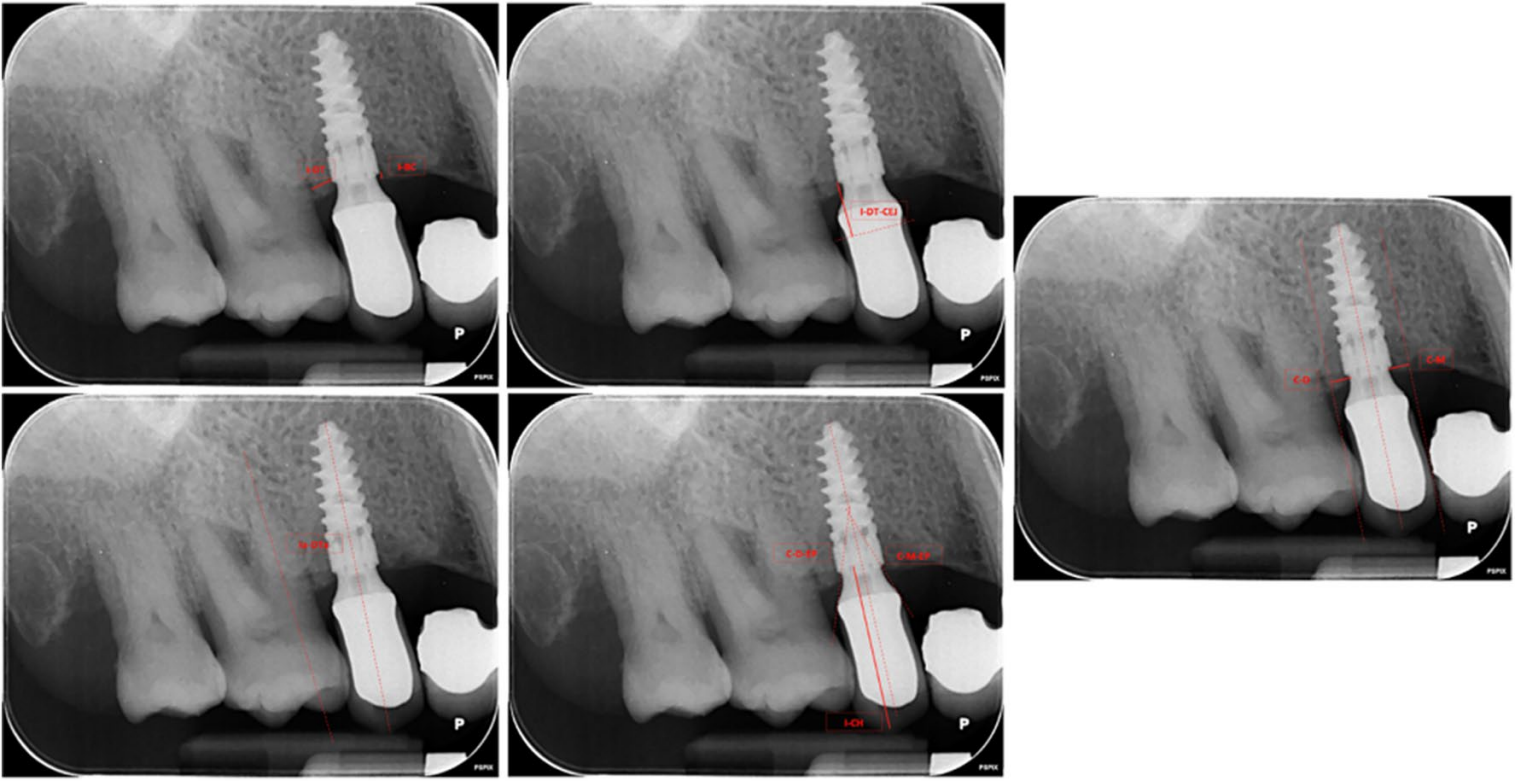
The figure shows, on a periapical radiograph, the parameters that were measured and the respective reference points

The following parameters were collected from clinical and radiographic records (Fig. 1):

- implant-related factors: implant type, length, diameter, vertical position of the implant (distance between implant neck and the bone level at the time of intervention (I-BC) (periapical radiograph)), distance between the implant neck margin and adjacent teeth (on periapical radiographs) (I-MT, I-DT [I-MI, I-DI]), distance between implant neck and the projection of CEJ of adjacent teeth (on periapical radiographs) (I-MT-CEJ, I-DT-CEJ), angle between the projection of the implant axis and the axis of adjacent teeth or implant.
- patient-related factors: age, gender, smoking status, presence of periodontal disease at the time of intervention / history of periodontal disease. The smoking status was assessed at the time of first implant placement, through a questionnaire. Following a previous study [27], the periodontal status was assessed before the implant placement and during each follow-up visit by following the criteria by Tonetti et al. [28].
- site-related factors: implant location
- prosthesis-related factors (to be evaluated using the radiographs taken after placement of the prosthesis): prosthesis type (single crown or partial fixed denture), fixation methods (screwed or cemented), platform switching, crown height (I-CH), extension of prosthetic cantilever (only for single-tooth restorations) (mesial and / or distal) (C-M, C-D), angle of emergence profile (mesial and distal) (C-M-EP, C-D-EP) which is calculated as the angle between the implant axis and the line tangent to the prosthetic crown [18], presence of misfit between the prosthesis itself and the abutment.

After diagnosing peri-implant status, clinical data were completely anonymized through the association of each subject to one identification code and the elimination of the document containing the link between them.

All radiographs considered in this study were taken using paralleling technique and using phosphor plate digital images with an exposure ranging from 0.16 to 0.22 s. The quality of the radiographs was appraised by adopting the criteria described in the inclusion criteria. Two previously calibrated operators (Cohen’s Kappa = 0.95 for diagnosis of peri-implantitis) (BM, SC) evaluated independently and in duplicate the radiographs for all the parameters. In case of disagreement between the two authors, (5% of the total cases) a third operator was involved (LF) and the disagreement was resolved by discussion. The linear radiographic measurements were taken by using the software ImageJ (Rasband, W.S., ImageJ, U. S. National Institutes of Health, Bethesda, Maryland, USA, https://imagej.nih.gov/ij/, 1997–2016.), and the mean values of the continuous measure was considered. When a high discrepancy (more than 30% difference) between the measures taken by the two operators was observed, the measure was re-taken jointly.

### Quantitative synthesis and statistical methods

The statistical analysis was carried out by using a dedicated professional software (SPSS, version 27, IBM) by one author (SC).

For sample size calculation we considered alpha = 0.05, power = 80% and we speculated a proportion between controls and cases of 3:1. We hypothesized to detect an effect of malposition with a proportion of 0.25 of exposed (i.e., peri-implantitis cases) in the control group and 0.5 in the test group. Considering a 10% rate of non-eligible records, we therefore decided to include 45 implants with peri-implantitis and 135 healthy implants. The proportion of exposed was estimated on the basis of the study published by Yi et al. in 2020 [19]. The sample was randomly selected, using the appropriate selection function, from the entire population of subjects responding to the inclusion criteria by using the software SPSS.

The normality of the distributions of the variables was initially assessed by Kolmogorov–Smirnov and Shapiro–Wilk tests. Descriptive statistics was then performed by presenting means, standard deviations, and confidence intervals (95% CI) for all continuous variables and frequencies for categorical variables.

The implant-level analysis of factors influencing the occurrence of peri-implantitis was performed through a multilevel multivariate logistic regression analysis (mixed). As indicated in a previously published study [29], the final tested model was made by an exploratory approach, in which each factor was tested individually in an empty model (the dependent variable was peri-implantitis) and the variables that were significant (*P* < 0.15) were included in a multivariate intermediate model after removing all non-significant factors. A final model was produced that included all factors that remained significant (*P* < 0.05).

In order to explore the role of those factors that resulted significant in the multi-level final model, an ancillary analysis (simple linear regression analysis) was also performed.

## Results

A total of 304 implants belonging to 114 subjects had complete clinical and radiographic data and were considered eligible for inclusion in the study. The clinical and radiographic records of a total of 180 implants from 90 subjects (57 females and 33 males) (mean 2.0 implants / subject) were randomly selected. Table 1 shows the main characteristics of the population. The mean age was 56.0 ± 11.8 years, 34 (37.8% of patients) had periodontitis (14 stage IV, 15 stage III, and 5 stage II), and 22 (24.4% of patients) were smokers at the time of implant placement. The mean follow-up was 6.4 ± 3.9 years, most of the implant-supported restorations were screw-retained (*n* = 107, 59.4%), and 116 implants (64.4%) were applied in multiple-unit prostheses. Malposition was recorded in 52 implants, namely 11 showing peri-implantitis and the remaining 41 belonging to the healthy group. Among the 11 malpositioned implants with peri-implantitis, 9 showed a distance < 1.5 mm from the adjacent tooth.

**Table 1:** Characteristics of the sample

No. subjects/no. implants	90 / 180
Females/males	57 (63.3%) / 33 (36.7%); in peri-implantitis group 19 (76.0%) / 6 (24.0%); in healthy group 38 (58.5%) / 27 (41.5%)
Maxillary / mandibular implants	89 (19 anterior, 70 posterior) / 91 (11 anterior, 80 posterior)^1^
Periodontitis / periodontally healthy	34 (37.8%) / 56 (62.2%); in peri-implantitis group 13 (52.0%) / 12 (48.0%); in healthy group 20 (30.8%) / 45 (69.2%)
Smokers / nonsmokers	22 (24.4%) / 68 (75.6%); in peri-implantitis group 6 (24.0%) / 19 (76.0%); in healthy group 16 (24.6%) / 49 (75.4%)
Mean age (years)	56.0 ± 11.8 [range: 27.9 – 80.0]
Mean follow-up (years)	6.4 ± 3.9 [range: 1.1 – 18.2]
Implant diameter (mm)	3.3: 6 / 3.3% 3.5: 16 / 8.9% 4.0: 85 / 47.2% 4.3: 67 / 37.2% 5.0: 6 / 3.3%
Implant length (mm)	8.0: 25 / 13.9% 10.0: 82 / 45.6% 11.0: 5 / 2.8% 11.5: 32 / 17.8% 13.0: 31 / 17.2% 15.0: 5 / 2.8%
Implant type / manufacturer	Nobel Biocare™ with moderately rough surface: 147 / 81.7% Dentsply implants™ with moderately rough surface: 33 / 18.3%
Prosthesis type	64 / 35.6% single-tooth implants 116 / 64.4% in fixed partial dentures
Fixation type	73 / 40.6% cemented 107 / 59.4% screw-retained
Malposition	35 / 19.4% (less than 1.5 mm distance with the adjacent tooth); in peri-implantitis group 9 (20.0%); in healthy group 26 (19.2%) 52 / 28.9% (less than 2-mm distance with the adjacent tooth); in peri-implantitis group 11 (24.4%); in healthy group 41 (30.4%)
Platform switching (no / yes)	99 (55.0%) / 81 (45.0%); in peri-implantitis group 30 / 15; in healthy group 69 / 66
Misfit (no / yes)	154 (85.6%) / 26 (14.4%); in peri-implantitis group 36 / 9; in healthy group 118 / 17

^1^Anterior means canines and incisors

The descriptive statistics of the implant- and prosthesis-related parameters is presented in Table 2.

**Table 2:** Measurements taken of the implant- and prosthesis-related parameters

Parameter	Peri-implantitis (mean ± SD [CI95%])	Without peri-implantitis (mean ± SD [CI95%])	All implants (mean ± SD [CI95%])
I-MT (mm)	3.73 ± 1.34 [3.10 – 4.35]	3.62 ± 1.88 [3.17 – 4.07]	3.64 ± 1.77 [3.28 – 4.01]
I-DT (mm)	3.34 ± 2.79 [1.57 – 5.12]	3.87 ± 2.60 [3.07 – 4.67]	3.75 ± 2.62 [3.04 – 4.46]
I-MI (mm)	5.41 ± 2.45 [4.32 – 6.49]	5.64 ± 2.84 [4.88 – 6.39]	5.57 ± 2.73 [4.96 – 6.18]
I-DI (mm)	5.32 ± 2.07 [4.37 ± 6.26]	5.07 ± 2.45 [4.45 – 5.69]	5.13 ± 2.35 [4.62 – 5.64]
I-MTa (mm)	4.58 ± 4.69 [2.44 – 6.71]	5.47 ± 3.64 [4.61 – 6.34]	5.27 ± 3.89 [4.46 – 6.08]
I-DTa (mm)	15.74 ± 17.31 [5.28 – 26.20]	12.77 ± 11.95 [8.89 – 16.64]	13.51 ± 13.36 [9.79 – 17.23]
I-MIa (mm)	5.10 ± 4.43 [3.08 – 7.11]	5.71 ± 4.33 [4.56 – 6.86]	5.54 ± 4.34 [4.56 – 6.52]
I-DIa (mm)	6.19 ± 7.31 [2.95 – 9.43]	6.95 ± 6.76 [5.25 – 8.66]	6.76 ± 6.87 [5.27 – 8.24]
I-MT-CEJ (mm)	3.80 ± 1.85 [2.96 – 4.64]	4.75 ± 1.89 [4.29 – 5.21]	4.53 ± 1.92 [4.12 – 4.93]
I-DT-CEJ (mm)	3.18 ± 2.11 [1.90 – 4.45]	3.47 ± 1.55 [2.98 – 3.95]	3.40 ± 1.69 [2.94 – 3.85]
I-CH (mm)	11.59 ± 2.13 [10.94 – 12.24]	11.70 ± 2.11 [11.34 – 12.06]	11.67 ± 2.11 [11.36 – 11.98]
C-M (mm)	3.02 ± 1.26 [2.64 – 3.40]	3.05 ± 1.47 [2.79 – 3.31]	3.04 ± 1.42 [2.83 – 3.25]
C-D (mm)	2.34 ± 1.37 [1.93 – 2.75]	2.38 ± 1.22 [2.17 – 2.59]	2.37 ± 1.26 [2.18 – 2.55]
Mesial emergence angle (degrees)	39.15 ± 13.81 [34.95 – 43,35]	35.85 ± 18.72 [22.64 – 39.06]	36,67 ± 17.65 [34.05 – 39.29]
Distal emergence angle (degrees)	32.79 ± 17.68 [27.48 – 38.11]	31.38 ± 18.05 [28.31 – 34.46]	31.74 ± 17.92 [29.10 – 34.38]
Number of implants having one emergence angle < 30°	9 / 45 (20.0%)	23 / 135 (17.0%)	32 / 180 (17.8%)
Number of implants having one emergence angle between 30° and 45°	17 / 45 (37.8%)	26 / 135 (19.3%)	43 / 180 (23.9%)
Number of implants having one emergence angle > 45°	19 / 45 (42.2%)	86 / 135 (63.7%)	105 / 180 (58.3%)

*SD* standard deviation, *CI95%* 95% confidence intervals

In the univariate analysis, the following parameters resulted significantly correlated to the occurrence of peri-implantitis: I-MT-CEJ, presence / history of periodontitis, presence of an emergence profile angle ≥ 45°, follow-up time, and follow-up time squared (Appendix Table 4). In the final multi-level model, the parameters that resulted significant were presence / history of periodontitis (OR = 5.945, CI95%: 1.093 – 32.334, *P* = 0.039) and presence of an emergence profile angle ≥ 45° (OR 9.094, CI95%: 2.017 – 40.995, *P* = 0.005) (Table 3).

**Table 3:** Results of multi-level analysis (final model)

	Null model		Multilevel final model		
Variable	OR	95% CI	OR	95% CI	*P*
Fixed					
Intercept	0.263	0.160 − 0.432	0.463	0.020 − 10.944	0.629
Presence / history of periodontitis					
No			*Reference*	*Reference*	*Reference*
Yes			5.945	1.093 − 32.334	**0.039**
Presence of angle of emergence profile ≥ 45°					
No			*Reference*	*Reference*	*Reference*
Yes			9.094	2.017 – 40.995	**0.005**
I-MT-CEJ			0.761	0.512 – 1.132	0.174
Follow-up			1.636	0.738 − 3.629	0.223
Follow-up^2^			0.965	0.916 − 1.016	0.173
Random					
Var (intercept)	1.793	0.871 − 3.691	1.661	0.412 − 6.706	
AIC	832.377		452.785		
BIC	835.542		455.154		

The bold entries represent the statistically significant *P* value

*OR* odds ratio, *CI* confidence intervals, *AIC* Akaike information criterion, *BIC* Bayesian information criterion

Regarding the factors being correlated to the emergence profile angle, the ancillary statistical analysis found that I-MT was significantly correlated to the angle of mesial emergence profile (*β* = 0.413, *P* < 0.001) as well as I-MT-CEJ (*β* =  − 0.280, *P* = 0.008), the extension of mesial cantilever C-M (β = 0.408, *P* < 0.001) and crown height (*β* =  − 0.177, *P* = 0.018). The angle of distal emergence profile was correlated to crown height (*β* =  − 0.202, *P* = 0.007), and on the extension of the distal cantilever (*β* = 0.397, *P* < 0.001). The details of the analysis are presented in Appendix Table 5.

## Discussion

The present retrospective case–control study failed to demonstrate that implant malposition, as defined by the analysis of periapical radiographs, could play a significant role in increasing the incidence of peri-implantitis in the selected cohort. However, the study found that history of periodontitis and the presence of a prosthetic emergence profile ≥ 45° are significantly correlated to an increased risk of peri-implantitis. Remarkably, the ancillary analysis conducted to understand how the emergence angle is influenced by implant position and prosthetic factors suggested that the distance between the adjacent tooth and the implant, as well as the apico-coronal position of the implant neck relative to the CEJ of the adjacent tooth are important factors influencing the emergence profile angle.

Our definition of “implant malposition” was based on the paper published by Buser and coworkers in 2004 [26], whose criteria were also adopted in the study on risk assessment for peri-implantitis by Canullo and coworkers [20]. First, we should underline that the criteria proposed for correct implant placement were initially proposed to optimize the esthetic outcomes, in particular in the anterior region of the maxilla, where such outcomes have a crucial importance [26]. In the present study we tested the hypothesis that such parameters could have an influence also in determining an increased risk of developing peri-implantitis (both in the anterior as well as posterior area). The study by Canullo et al. [20] evaluated implant malposition by performing measurements on intraoral photographs in a cohort that mainly presented posterior implants (223 out of 332 implants), and they reported that the Odds Ratio for peri-implantitis related to implant malposition was 48.2 (11.4 – 204.1). Remarkably, in that study only two (out of 42) of the implants showing malposition were healthy at the time of the examination [20].

On the contrary, the present study found contrasting results, as no significant correlation was identified between malposition and occurrence of peri-implantitis. This unforeseen outcome could be due to several factors, including the different methods applied for determining the distances, the choice of statistical analysis and the characteristics of the sample (e.g., we excluded full-arch restorations).

The issue of implant malposition should be further explored in future clinical studies, as well as the effect of the experience of the operators (namely the surgeons) on implant survival over time. It is recommended that similar criteria to define malposition should be applied in future studies on the same topic, as this would allow to make meaningful comparisons between outcomes. Remarkably, one recently published systematic review of the literature reported that the experience of the surgeon (based on the number of implants placed before the intervention) was a significant factor influencing the outcomes, being implants placed by more expert surgeons (who have placed more than 50 implants) less prone to failure (OR = 2.18) [21].

In our study the operators’ experience was not evaluated and this could be considered a limitation. However, based on our findings, the experience of prosthodontists and of dental technicians may be of relevance on the outcomes of implant-supported restorations. Indeed, it is known that the characteristics of the prosthetic restoration may have an influence on the cleansibility and on the possibility of maintaining a high level of oral hygiene, thus leading to satisfactory clinical outcomes over time [30]. In the present research we found that having an emergence angle ≥ 45° significantly increased the incidence of peri-implantitis (OR = 9.094), whilst we found no statistically significant evidence when applying other threshold values, such as 30° and 20°. This outcome is in partial agreement with what described in the literature. In particular, the study by Katafuchi and coworkers on 168 implants (83 subjects) reported a higher prevalence of peri-implantitis in the bone-level group having an emergence angle > 30° than [18]. Differently from our study, they used the 2012 definition of peri-implantitis, and they tested only 30° as threshold value. Notably, they found no correlation between emergence angle and peri-implantitis in tissue-level implants. Another research group evaluated the association of prosthetic factors, such as emergence profile, emergence angle and crown / implant ratio with peri-implantitis on 349 implants (169 patients) [19]. In that study, they adopted 30° as threshold value, reporting a significantly higher percentage of peri-implantitis in the group that showed an emergence angle of > 30° (OR = 3.80, CI95%: 1.75 – 8.22, *P* < 0.05). Moreover, they found that the risk of peri-implantitis raised in a statistically significant manner with the increase of the emergence angle.

In summary, the findings of our research, confirmed that so-called “over-contoured” prosthetic restorations are more prone to develop peri-implantitis, probably because of the limitations in maintaining oral hygiene, thus confirming the outcomes of previous studies [31, 32]. However, our data showed that the presence of a misfit may not be correlated to clinical complications such as peri-implantitis, and it could be considered as a minor factor as compared to the influence of the characteristics of the prosthesis emergence angle, thus corroborating the findings of a previous systematic review [33].

There is strong evidence that periodontitis (and history of periodontitis) is an independent risk factor for peri-implantitis [34]. In our study, the multi-level analysis showed that periodontitis increased up to six-fold the risk of developing peri-implantitis, thus stressing the importance of patient selection and the need of paying extra attention when planning and performing implant-supported restoration in periodontitis patients.

We also performed an ancillary analysis on the available data to understand if there was a correlation between the various parameters collected and the emergence angle, which we identified as a factor influencing the occurrence of peri-implantitis. To the best of our knowledge, this is the first study that evaluated it. The distance between the implant neck and the adjacent tooth (in our cases, the mesial one) was obviously correlated to the extent of mesial cantilever and it was statistically correlated to the mesial emergence angle (i.e., the higher the distance, the higher the angle) for geometrical reasons. For the same geometrical reasons, the higher the distance between the implant neck and the CEJ of adjacent tooth (mesial) and the higher was the crown height, both being correlated to a decrease of the emergence angle. Distal emergence profile presented a similar pattern of correlations, that was indeed limited by the fact that some implants did not present a distal element, either a tooth or an implant. While in several studies the issue of implant-implant or implant-tooth distance was explored in relation to esthetic outcomes, including the presence / absence of peri-implant papilla [35–37], in the present study we did not find a direct effect of such distance on the occurrence of peri-implantitis but it was probably a co-factor, by influencing the emergence angle and thus, the possibility of maintaining oral hygiene. Such hypothesis needs to be supported by future research in the field, but it appears corroborated by our preliminary data.

The external validity of the present results could have been influenced by some weaknesses of the study protocol that deserve to be discussed. Firstly, the sample size is lower than in other studies previously published on the same topic, although an accurate sample size calculation had been performed. We included patients that presented for maintenance visits (at least yearly) but we did not examine analytically the data about the level of oral hygiene, which is a known risk factor [14, 38]. Furthermore, all the measures and, consequently, the assumptions from the statistics were made based on periapical radiographs. Even though the same was done in all other previously published papers, we should assume that bidimensional radiographs not performed with an individualized holder might present a certain level of distortion and this could lead to potentially inaccurate linear measurements, particularly in certain sectors of the mouth. Indeed, as reported in the paper by Wakoh and colleagues, measurements made in periapical radiographs could be very accurate in molar region but less accurate in position with higher curvature (such as canine / premolar area) [39]. Nevertheless, in all patients the parallel cone technique was applied, and we performed a qualitative evaluation of the x-rays by using standardized criteria. Moreover, in vitro and ex vivo studies confirmed the overall reliability of periapical radiographs for linear measurements involving dental implants [40, 41]. The use of bidimensional radiographic imaging should also be considered as a limitation of the study, even though justified by its routinary use in standard follow-up visits. Another limitation of the study includes the fact that the definition of malposition refers to radiographic parameters only and does not consider the characteristics of the soft tissues around implants, like for instance the residual keratinized mucosa after prosthetic finalization. Moreover, we analyzed the smoking status as recorded at the time of implant positioning, but the assessment of the same parameter at the time of the diagnosis of peri-implantitis may be useful for further analyses. Finally, a recent paper reported that the case definition we used for peri-implantitis in the present study may present high level of specificity but relatively low sensitivity [42].

Considering all the aforementioned, the intrinsic limitations coming for retrospective data, and the relatively broad range of follow-ups, our results should be interpreted with caution and need to be confirmed by future studies. It is also important to note that all the included implants presented with a moderately rough surface, hence our conclusions may not necessarily be generalized to implants with a different surface.

Despite the limitations discussed above it can be concluded that it was not possible to find a significant correlation between implant malposition (as defined before) and the occurrence of peri-implantitis. Nonetheless, a ≥ 45° emergence angle of the prosthetic restoration could be recognized as an independent risk factor for peri-implantitis, as well as history of periodontitis. The angle of prosthetic emergence should be considered as dependent on some positional characteristics of the implant itself, in relation to surrounding teeth and implants and this parameter should be carefully considered during implant planning. Based on our results, it can therefore be concluded that a meticulous prosthetic planning should be performed to avoid over-contoured restorations. In addition, during implant placement it is fundamental for the surgeon to consider carefully the distance of the implant from the adjacent tooth/implant and the apico-coronal position of the implant neck relative to the CEJ of the adjacent tooth, since the emergence profile would be directly influenced by these parameters.

More studies, both retrospective and prospective, on larger samples, based also on tridimensional evaluations are warranted to better understand how and if implant position could play a role in the development of peri-implantitis.

## Appendix

Table 4

**Table 4:** Results of univariate analysis (outcome: occurrence of peri-implantitis)

Parameter	Exp	CI 95%	Sign
Follow-up	1.684	1.042 – 2.723	0.034
Follow-up^2^	0.978	0.951 – 1.005	0.108
Periodontitis / history of periodontitis	2.210	1.155 – 9.119	0.026
Smoking	1.034	0.314 – 3.408	0.956
I-MT	0.784	0.449 – 1.369	0.381
I-DT	1.134	0.755 – 1.704	0.533
I-MI	1.011	0.765 – 1.338	0.934
I-DI	1.113	0.727 – 1.704	0.610
I-MTa	1.054	0.788 – 1.410	0.713
I-DTa	1.029	0.963 – 1.099	0.387
I-MIa	0.872	0.652 – 1.166	0.344
I-DIa	1.033	0.929 – 1.149	0.533
I-MT-CEJ	0.477	0.184 – 1.234	0.102
I-DT-CEJ	0.991	0.420 – 2.338	0.982
I-CH	0.903	0.725 – 1.124	0.357
C-M	0.864	0.621 – 1.202	0.382
C-D	1.038	0.737 – 1.462	0.828
Mesial emergence angle	0.993	0.968 – 1.019	0.612
Distal emergence angle	0.985	0.962 – 1.010	0.243
Platform switching	0.716	0.279 – 1.838	0.485
Misfit	0.692	0.258 – 1.859	0.463
Emergence angle < 20°	2.571	0.346 – 19.104	0.354
Emergence angle < 30°	0.851	0.158 – 4.598	0.851
Emergence angle < 45°	0.382	0.139 – 1.047	0.061
Malposition (1.5 mm)	0.621	0.097 – 3.989	0.614
Malposition (2.0 mm)	1.694	0.314 – 9.142	0.538

Table 5

**Table 5:** Results of regression analysis exploring the factors influencing the emergence angle

Parameter	*β*	Sign
Mesial emergence angle		
I-MT	− 0.134	0.390
I-MTa	0.080	0.378
I-MT-CEJ	0.259	0.043
I-CH	0.164	0.197
C-M	− 0.431	0.006
Distal emergence angle		
I-DT	− 0.225	0.269
I-DTa	0.383	0.039
I-DT-CEJ	0.126	0.429
I-CH	0.291	0.068
C-D	− 0.473	0.002
