# Correction to: The influence of implant position and of prosthetic characteristics on the occurrence of peri‑implantitis: a retrospective study on periapical radiographs

**DOI:** 10.1007/s00784-023-05373-9

**Published:** 2023-11-10

**Authors:** Stefano Corbella, Benedetta Morandi, Elena Calciolari, Alice Alberti, Luca Francetti, Nikolaos Donos

**Affiliations:** 1 Department of Biomedical, Surgical and Dental Sciences, Universita degli Studi di Milano, Milan, Italy; 2 IRCCS Ospedale Galeazzi Sant’Ambrogio, Milan, Italy; 3 Centre for Oral Clinical Research, Institute of Dentistry, Faculty of Medicine and Dentistry, Queen Mary University of London, London, UK; 4 Centro di Odontoiatria, Dipartimento di Medicina e Chirurgia, Universita di Parma, Parma, Italy

**Correction to: Clinical Oral Investigations**

**https://doi.org/10.1007/s00784-023-05303-9**

In the published paper, the author Nikos Dono should be Nikolaos Donos.

The original article has been corrected.

